# Complete chloroplast genome sequence of *Micranthes melanocentra* (Saxifragaceae)

**DOI:** 10.1080/23802359.2022.2069523

**Published:** 2022-05-03

**Authors:** Jianfang Li, Zhan-Lin Liu

**Affiliations:** Key Laboratory of Resource Biology and Biotechnology in Western China (Ministry of Education), College of Life Sciences, Northwest University, Xi’an, China

**Keywords:** *Micranthes melanocentra*, plastome, phylogeny

## Abstract

The phylogenetic relationships among *Micranthes* taxa remain unclear due to their diversification. Here, we report the complete chloroplast genome of *Micranthes melanocentra* obtained using high-throughput sequencing technology to provide genomic information for phylogenetic analyses. The plastome is 155,317 bp, with a large single-copy region (LSC) of 86,784 bp, a small single-copy region (SSC) of 18,007 bp, and a pair of 25,263 bp inverted repeat regions (IRs). The genome contains 132 genes, including 86 protein-coding genes, 37 tRNA genes, 8 rRNA genes, and 1 pseudogene. The GC content of the plastome is 37.9%; corresponding values in the LSC, SSC, and IR regions are 36.1%, 31.9%, and 43.3%, respectively. The phylogenetic tree supports the separation of *Micranthes* from *Saxifraga s.l*.

The genus *Micranthes* Haworth (Saxifragaceae) initially belonged to *Saxifraga* L. The genus was identified as a monophyletic group in molecular phylogenetic analyses (Gornall et al. 2000; Tkach et al. 2015), comprising 79–90 species, which are native to subarctic and subalpine regions of the Northern Hemisphere. It is becoming an ideal model for investigating the diversification, adaptation, and evolutionary history influenced by the changing climate in montane and Arctic biomes (Stubbs et al. 2019). However, infrageneric relationships have not been comprehensively explored because of polyploidization, hybridization, rapid radiation, and/or niche shifts (Ebersbach et al. 2017). With the development of high-throughput sequencing technology, complete chloroplast genomes have been obtained and are valuable in phylogenetic analyses of complex plant taxa. *Micranthes melanocentra* (Franchet) Losinskaja 1896 is distributed at an altitude of 3000–5300 m in western China and the eastern Himalayas (Wu et al. 2001). *Micranthes melanocentra* harbors polyphenolic compounds that protect against NS3 serine protease activity of hepatitis C virus (Zuo et al. 2005). As a traditional Chinese herbal medicine, *M. melanocentra* is also used to treat eye and biliary diseases (Luo and Tian 1997). Here, we determined the chloroplast genome of *M. melanocentra* to provide useful genomic information for phylogenetic studies of the genus *Micranthes*.

Fresh leaves of *M. melanocentra* were collected from the Qinling Mountains in China (N33.99°, E107.80°) and dried with silica gel. A specimen was deposited at the herbarium of Northwest University (contact person: Zhan-Lin Liu, liuzl@nwu.edu.cn) with voucher number 20180375. DNA sequencing was performed using an Illumina HiSeq2000 (Novogene Co. Ltd.). Chloroplast genome assembly was performed using GetOrganelle v1.73 (Jin et al. 2020) and annotated using Geneious v9.0 (Kearse et al. 2012) with *Saxifraga stolonifera* (NC_037882) as the reference.

The chloroplast genome of *M. melanocentra* (MT740256) is 155,317 bp in length and has a large single-copy region (LSC) of 86,784 bp, a small single-copy region (SSC) of 18,007 bp, and a pair of inverted repeat regions (IRs) of 25,263 bp. The genome contains 132 genes, including 86 protein-coding genes, 37 tRNA genes, and 8 rRNA genes. One pseudogene (rps19) is located at the IR-LSC junction. Sixteen genes were duplicated in the IRs, including 5 protein-coding genes (*rpl2*, *rpl23*, *ycf2*, *ndhB*, and *rps7*), 7 tRNA genes, and 4 rRNA genes. 16 genes contain a single intron, and 3 genes (*ycf3*, *rps12*, and *clpP*) harbor two introns. The overall GC content of the LSC, SSC, and IR regions are 36.1%, 31.9%, and 43.3%, respectively.

To elucidate the position of *M. melancenotra* in Saxifragaceae, 19 complete chloroplast genomes were used as representative genomes to construct phylogenetic relationships. RaxmlGUI v2.0 (Edler et al. 2021) was performed using the maximum-likelihood method with *Rhodiola rosea* (MH410216) and *Sedum sarmentosum* (JX427551) as the outgroups. Phylogenetic analysis showed that *M. melancenotra* was clustered together with *Chrysosplenium*, rather than species in *Saxifraga* (Figure 1). The finding supported the separation of *Micranthes* from *Saxifraga* sensu lato, as proposed by Tkach et al. (2015). This result was further confirmed by a phylogenetic tree constructed using partial gene sequences of *matK*, one of the core DNA barcodes for angiosperm phylogeny (Supplementary Figure 1).

**Figure 1.**
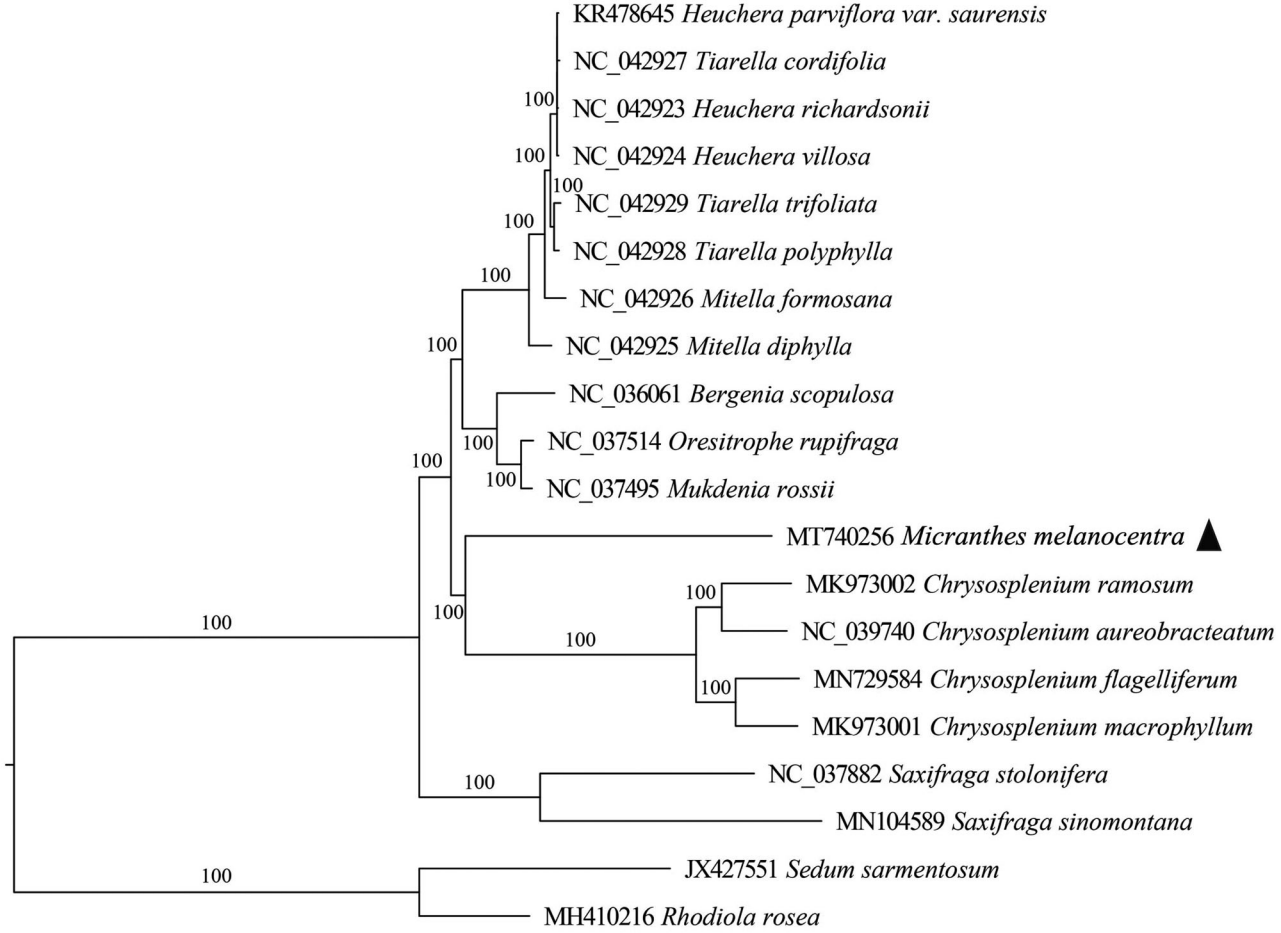
Maximum-likelihood (ML) tree based on the complete chloroplast genomes in Saxifragaceae with Crassulaceae as outgroup. Numbers near nodes are bootstrap support values based on 1000 replicates.

The plastome sequence of *M. melancenotra* reported here provides a valuable genomic resource for the phylogenomic analyses of Saxifragaceae.

## Ethical approval

Ethical approval is not applicable to the study.

## Authors’ contributions

Li J analyzed the data and wrote the draft of the paper. Liu Z-L designed the experiments and revised and approved the final version of the manuscript. All authors agree to be accountable for all aspects of the work.

## Supplementary Material

Supplemental Material

## Data Availability

The genome sequence data that support the findings of this study are openly available in GenBank of NCBI at (https://www.ncbi.nlm.nih.gov/) under the accession no. MT740256. The associated BioProject, SRA, and Bio-Sample numbers are PRJNA720175, SRX10528772, and SAMN18642400, respectively.
