# Supplementary figures and images for: Complete chloroplast genome sequence of Micranthes melanocentra (Saxifragaceae)
Source: Mitochondrial DNA B Resour. 2022 May 3;7(5):761–3. doi: 10.1080/23802359.2022.2069523 (PMC9068006; doi:10.1080/23802359.2022.2069523)

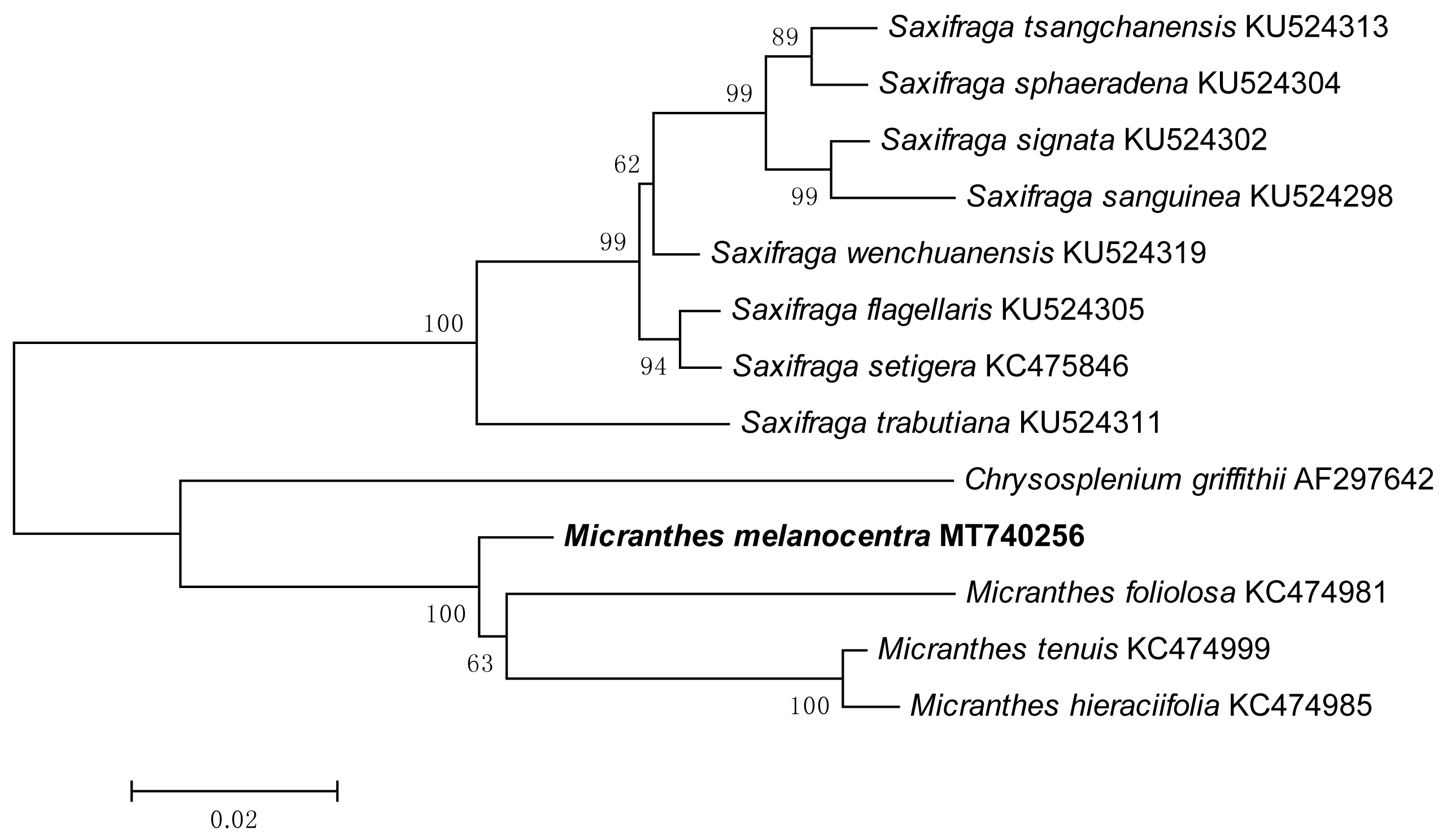

Supplement: Supplemental Material [file TMDN_A_2069523_SM6887.jpg]
